# Microplastic Pollution Focused on Sources, Distribution, Contaminant Interactions, Analytical Methods, and Wastewater Removal Strategies: A Review

**DOI:** 10.3390/ijerph19095610

**Published:** 2022-05-05

**Authors:** Sílvia D. Martinho, Virgínia Cruz Fernandes, Sónia A. Figueiredo, Cristina Delerue-Matos

**Affiliations:** REQUIMTE/LAVQ—Associated Laboratory for Green Chemistry (LAQV) of the Network of Chemistry and Technology (REQUIMTE), Instituto Superior de Engenharia do Porto—Instituto Politécnico do Porto, Rua Dr. António Bernardino de Almeida, 431, 4249-015 Porto, Portugal; silviadpmartinho@gmail.com (S.D.M.); cmm@isep.ipp.pt (C.D.-M.)

**Keywords:** microplastics, water, wastewater, analytical methods, adsorption

## Abstract

Plastics have been one of the most useful materials in the world, due to their distinguishing characteristics: light weight, strength, flexibility, and good durability. In recent years, the growing consumption of plastics in industries and domestic applications has revealed a serious problem in plastic waste treatments. Pollution by microplastics has been recognized as a serious threat since it may contaminate all ecosystems, including oceans, terrestrial compartments, and the atmosphere. This micropollutant is spread in all types of environments and is serving as a “minor but efficient” vector for carrier contaminants such as pesticides, pharmaceuticals, metals, polychlorinated biphenyls (PCBs), and polycyclic aromatic hydrocarbons (PAHs). The need to deeply study and update the evolution of microplastic sources, toxicology, extraction and analysis, and behavior is imperative. This review presents an actual state of microplastics, addressing their presence in the environment, the toxicological effects and the need to understand their extent, their interactions with toxic pollutants, the problems that arise in the definition of analytical methods, and the possible alternatives of treatments.

## 1. Introduction

### 1.1. Microplastics

Plastics have been one of the most used materials in the world. Over the last century, humanity has learned how to create different types of plastics that are stronger, lighter, and more flexible than previous materials. Synthetic polymers, currently known as plastics, can be produced either from natural substances, such as cellulose, or, more often, from petroleum and other fossil fuels [1]. Due to the uncountable applications of plastics and their widespread use, they have become a source of pollution all over the world.

For a better understanding of this emergent problem, there is a need to identify the sources of plastics, the quantity that is spread, and the methods to treat all the environmental compartments, to prevent future dispersion, and to understand the different effects that they may provoke on living organisms and humans’ health.

In 2019, global plastic production reached 370 million tons, and European plastic production reached almost 58 million tons. In 2020, in Europe, 39.6% of plastics were used for packing and 20.4% were used for building and construction, these being the areas that consume the most. In recent years, methods of recycling materials have been developed; however, only 32.5% of plastics go to recycling, 42.6% are used in energy production, and 24.9% are sent to landfills [2]. Even though recycling has been growing in recent years, it is important to know that approximately 50% of plastics have single-use disposable applications, which leads to an accumulation of plastics in the environment. Single-use plastics are used more in packing, agricultural films, and disposable consumer items. Only 20 to 25% of plastics are used in products for long-term use, such as pipes, cable coatings, and structural materials, and the remaining plastics are used for products with intermediate lifespans, such as electronic goods, furniture, and vehicles [1].

The lifetime of plastics is a concern since, on one hand, their good durability allows extended use and renders them a good material choice, but on the other hand, it has been proved that the resistance to degradation of plastic waste is problematic [3]. Currently, there is legislation in countries in order to minimize the impact of plastics; however, the environment has been suffering from the accumulation of plastic waste. It is estimated that 10% of plastics produced end up in the oceans, where they persist, accumulate, and are a way of transporting pollutants [3]. It is estimated that 95% of marine waste has plastics as the main component [4].

### 1.2. Sources of Microplastics

In recent years, an increasing environmental concern has been reported about “microplastics”: tiny plastic particles smaller than five millimeters (5 mm), with two main types of sources, primary and secondary sources [3].

“Primary microplastics” are defined as plastic pieces under 5 mm, which corresponds to the size that they are manufactured for in a particular industry or domestic applications [5]. There is a huge variety of products that are used in daily care that contribute to the increase in the waste of microplastic particles in the environment, such as facial cleansers, toothpaste, resin pellets, and cosmetics (e.g., shower gels, scrubs, peelings, eye shadow, deodorants, blush powders, makeup foundation, mascara, shaving cream, baby products, bubble bath lotions, hair coloring, nail polish, insect repellents, and sunscreens) [1,6,7,8]; others are used in air blasting [1]. “Open use” of these products is one of their characteristics, and then they are washed off and end up in drains [5]. Their use in medicine as vectors for pharmaceuticals has been increasingly reported [1]. Microplastics are also used in air blasting technology, involving the processes of blasting acrylic, melamine, or polyester microplastic scrubbers at machinery, engines, and boat hulls to remove the rust and paint [1].

“Secondary microplastics” are the result of the breakdown of larger plastic particle waste. The physical, biological, and chemical degradation processes can reduce and change the structural integrity of the plastic, resulting in fragmentation [5]. There are numerous environmental factors such as sunlight and temperature that can cause plastic fragmentation, and size and density changes [1]. Ultraviolet radiation of sunlight can promote the oxidation of the microplastic matrix, which leads to the cleavage of bonds [1,5]. The fragmentation of macroplastics into microplastics is more active on beaches due to the influences they face, such as the high ultraviolet radiation of the sun, available oxygen, and physical abrasion by waves, sand, and wind [1,5].

Independent of the source, primary or secondary, it is known that the marine environment hosts approximately 92% of global marine plastic debris [9], and microplastics were reported with a concentration of 102,000 particles per cubic meter in seawater [10]. The consequences of this global exposure to microplastics are not well understood, so the goal should be to understand the impact of microplastics’ presence and define several methods that work for any amount of microplastics spread into the environment, as well as the development of new techniques to prevent this accumulation in the environment.

### 1.3. Microplastics in the Aquatic Environment

The first studies reporting the presence of plastics in oceans were in the 1970s but did not attract the attention of the scientific community [11,12,13]. In the following decades, when researchers started to see the accumulation and impact of plastic dispersion on ecosystems, this topic started to receive increasing interest. Recent reports estimated that plastic litter contributes to 80% of the plastic found in marine litter, being a consequence of incorrect disposal of plastic waste [1]. Microplastics used in the primary industries, cosmetics and air blasting, are extensively used by the world’s population. The incorrect disposal of plastics can represent a high potential path to end up in the marine environment via rivers and wastewater systems [14]. However, several sources contribute directly to the increase in plastics in the oceans, such as coastal tourism, recreation and commercial fishing, marine vessels, and marine industries. Tourism and recreational activities, unfortunately, are associated with the plastics discharged along beaches and coastal resorts and their negative impact [15]. Activities related to fishing are also reported as a direct contribution to plastic marine pollution, being estimated to represent 18% of the marine plastic debris in the oceans, since fishing gear, monofilament line, and nylon netting are commonly found discarded or lost [16].

### 1.4. Microplastics in the Terrestrial Environment

Several studies [1,3,17,18,19] have shown that terrestrial systems have also been affected by the unconscious disposal of plastics. It was estimated that there may be 4 to 23 times more microplastics in the soil than in the oceans, representing a significant environmental risk [17]. Microplastics have three major types of sources in terrestrial compartments: inputs from agricultural practices, the influence of runoff, and the degradation of large plastics after improper disposal. In the agriculture practice, there are a few processes that can contribute to the disposal of microplastics in the soil, such as the use of sewage sludge as a fertilizer. Several studies [17,18,19,20] believed that the majority of microplastics are concentrated in the solid phase of sludge. There are a few sources that contribute to the presence of microplastics in sludges such as the microbeads used in cosmetics products and industry, fibers released from the washing of synthetic clothes, tire debris, and fragmented plastics from urban runoff [21]. Additionally, in agriculture, some techniques used to increase crop yields and reduce pests may contribute to the presence of microplastics. The use of plastic mulching is common, and regularly there are plastic films left behind after use, causing an accumulation of plastics in soils, which will suffer transformations, such as fragmentation and degradation, that lead to the formation of different sizes of plastics, from micro- to nanoplastics [21].

## 2. Microplastics’ Interaction with Other Contaminants

Microplastics are so ubiquitous on our planet that there are no places free of their pollution, and their presence has been demonstrated in surface waters [22], seabed sediments [23], beaches [22], soils [17,21], freshwaters [24,25], wastewater effluents [22], air [26], sea ice in the Arctic and Antarctic regions, several species of living organisms, food products (e.g., salt and honey), bottled water, and indoor and outdoor air [22,27]. They are now considered a threat to the equilibrium of the environment and human health since they can act as vectors for environmental contaminants [17]. The characteristics of microplastics, such as their composition, size, and potential to adsorb/release toxic and endocrine-disrupting compounds, have been investigated [28,29,30]. The global dispersion of these micropollutants in the marine environment has been widely explored, and it has been recognized as one of the most impacting threats, since it affects the equilibrium of ecosystems [31]. Agriculture, industry, energy production, and human consumption use freshwater as the main source of water. The presence of microplastics has been reported in freshwaters, turning these into an easy route of contamination of agricultural lands and industrial products [24,32]. Further, in this review, the efficiency of WWTPs in the removal of microplastics is discussed. Ofori et al. [33] described the benefits and challenges of reusing treated wastewater, focusing on the soil impact. The quality of treated wastewater used for irrigation will be essential to maintaining and improving the quality of the soil [33].

The capacity to sorb and accumulate organic and inorganic contaminants can be a severe environmental problem since some contaminants can be released and find entrance into organisms [21,34]. Studies have demonstrated that microplastics can sorb various contaminants, such as polycyclic aromatic hydrocarbons (PAHs), polychlorinated biphenyls (PCBs), dioxin-like chemicals, polybrominated diphenyl ethers (PBDEs), toxic metals, pharmaceuticals, pesticides, and herbicides [21,34,35].

It is important to develop research and techniques to study the chemical/physical interactions between microplastics and contaminants, using the theoretical knowledge of the sorption behavior of the microplastics. According to published studies, strong interactions may occur between contaminants and the surface of microplastics, promoting the adsorption of the contaminant through physical (e.g., electrostatic) or chemical interactions, which are favored by the contaminant hydrophobicity [36].

The sorption and desorption kinetics are a starting point in the laboratory to understand the behavior of plastic particles as a vector [37]. Therefore, depending on the extension of the damages prompted by the presence of microplastics, they may lead to considerable alterations in the natural function of ecosystems.

Chemical and physical interaction studies between different contaminants and microplastics have been explored, during or after their release into the environment, and this can be a way of understanding the global behavior of microplastics. The determination of particle-bound contaminants and the study of the sorption behavior of microplastics, using theoretical models with increased complexity, improve our knowledge of the role of vectors that microplastics can represent.

Sorption is the process of transferring chemicals from a fluid phase such as air or water to a solid phase. This term includes two different interactions: adsorption and absorption. The adsorption process involves a wide range of different interaction forces, such as van der Waals, ionic, and steric forces, л–л interactions, and covalent bonds, concerning the sorbent’s surface [38]. For a low concentration of contaminants in the environment, the adsorption process will lead to stronger interactions between the forces involved on the surface of the adsorbent. However, if the concentration of the contaminant is higher, the absorption process will occur once there is a great volume available to settle the molecules [38,39]. Several factors can influence the adsorption dynamics such as the hydrophobic properties of the sorbate, the properties of the solid phase (microplastic), and the surface-to-volume ratio of the solid [37,39]. Properties such as the polymer size, shape, and density can be also considered for the sorption process [37]. The adsorption process can be divided into physisorption and chemisorption according to the association between the adsorbate and adsorbent. The physical sorption process occurs for a difference in energy and forces of attraction, denominated by van der Waals forces, fixed by the adsorbent molecules. On the other hand, the chemical sorption process promotes the rearrangement of the molecules, changing their orbitals, like a chemical reaction [40]. Usually, chemical sorption is an irreversible process except when the covalent bonds are broken, leading to a desorption process of the chemicals from the solid phase [37]. To achieve and understand these behaviors, it is important to perform kinetic and equilibrium studies.

Studies were reported exploring the interactions between different types of contaminants such as PAHs, PCBs, dioxin-like chemicals, PBDEs, toxic metals, pharmaceuticals, pesticides, and herbicides and microplastics in aquatic environments [21,34,35]. These studies also showed the analytical methodologies and the experiment conditions, such as the concentrations of the contaminants and the adsorbents. Pesticides, pharmaceuticals, and metals can be easily absorbed by microplastics, and this process can promote an increased ecotoxicological risk since these contaminants are laden with high levels of pollutants and can be ingested by different living organisms. In Table 1, several kinetic studies are summarized concerning the sorption of different groups of contaminants (e.g., pesticides, pharmaceuticals, metals) by microplastics and their experimental conditions. The lack of standard procedures in sample preparation and analysis and the use of different experimental conditions are some of the major problems in this area, making it difficult to compare studies.

Polyethylene (PE), polystyrene (PS), polypropylene (PP), polyamides (PA), and polyvinyl chloride (PVC) are the five microplastics more frequently found in the aquatic environment [41]. According to different studies, researchers have been focused on the adsorption studies of different environmental contaminants (e.g., pesticides in agriculture, pharmaceuticals in wastewaters, metals) in these microplastics [42,43,44,45,46,47,48,49,50]. Kinetic and equilibrium models were used in these studies, being fitted to the experimental results, to estimate the adsorption capacity for different contaminants of microplastics. According to the published studies, mentioned in Table 1, it is possible to understand the enormous possibilities of interactions between microplastics and contaminants. Regardless of the different experimental conditions (concentrations and particle size), all the microplastics mentioned can adsorb different contaminants. Pesticides, pharmaceuticals, personal care products, and metals are adsorbed by microplastic particles and may be transported to different ecosystems. The adsorption capacity of microplastics has been studied, and significant adsorption capacities were observed. The three different groups of contaminants, pesticides, pharmaceutical and personal care products, and metals, present different behaviors in the presence of different microplastics. The properties of microplastics directly influence the adsorption process (e.g., polymer size, shape, and density, hydrophobic properties, and the surface-to-volume ratio). The comparison of the different studies is a starting point to understanding the behavior of microplastics in various conditions. The higher the adsorption capacity for a given pollutant of a specific microplastic, the higher the harmful potential of the consortium microplastic/pollutant. With PE, PP, and PS being the most commonly detected microplastics in the environment, their adsorption behavior has been explored [51].

According to Table 1, PE presents the highest adsorption capacities. For pesticides, it was possible to observe the minimum adsorption capacity (2.87 μg g^−1^) for Dipterex and the maximum for Difenoconazole (273 μg g^−1^). For pharmaceutical and personal care products, the highest adsorption capacity was observed for Sulfamethoxazole (660 μg g^−1^) and Ciprofloxacin (5850 μg g^−1^). PE adsorption capacity shows a variation from 0.0101 μg g^−1^ for Cd to a maximum of 4700 μg g^−1^ for Cr.

For pesticides (Table 1), PP presents the minimal adsorption capacity of 0.81 μg g^−1^ for Imazapic and a maximum of 62.7 μg g^−1^ for Fipronil. For pharmaceutical and personal care products, the lowest adsorption capacity was obtained for Tysolin (3333 μg g^−1^) and the highest for Sulfamethoxazole (6900 μg g^−1^). For metals, the lowest adsorption capacity was verified for Cr, with a capacity of 624 μg g^−1^, and the highest for Pb, with 5550 μg g^−1^.

PS presented a minimum adsorption capacity of 0.208 μg g^−1^ for Triadimenol and the highest capacity for Fipronil with 50.8 μg g^−1^, from the presented pesticides (Table 1). For pharmaceutical and personal care products, the lowest adsorption capacity was observed for Cephalosporin-C (710 μg g^−1^), and the maximum adsorption capacity was obtained for Sulfamethoxazole (114,000 μg g^−1^). Regarding metals, Cu presented the lowest adsorption capacity (8.46 μg g^−1^) and Pb the highest (2940 μg g^−1^).

Comparing the adsorption capacity of the microplastics presented in Table 1, pharmaceutical and personal care products present the highest values, between 660 and 96,400 μg g^−1^, followed by metals, in the range from 0.01 to 5550 μg g^−1^, and pesticides, in the range from 0.061 to 333 μg g^−1^.

These studies (Table 1) were compared to provide a general idea of the interactions between microplastics and contaminants. In general, it is shown that aged microplastics present a higher capacity to adsorb contaminants when compared to virgin microplastics. In future works, it is important to understand the influence that environmental conditions and microplastic aging may have as an approach to real conditions.

**Table 1:** Summary of sorption studies (kinetics and equilibrium) for different systems of microplastics/contaminants (pesticides, pharmaceutical and health care products, and metals).

Microplastic Type	Particle Size (μm)	MP Concentration (g/L)	Contaminant	Contaminant Concentration (μg/L)	Adsorption Capacity (Qm) (μg/g)	Analytical Methods	References
**Pesticides**							
High-density polyethylene (HDPE)	40–48	10	Epoxiconazole Tebuconazole Myclobutanil Azoxystrobin Simazine Terbuthlazine Atrazine Metolachlor	100	0.061–0.963	Ultra-high performance liquid chromatography (UHPLC)	[52]
Polyethylene (PE)	>5	10	Carbendazim Dipterex Diflubenzuron Malathion Difenoconazole	1000	4.44 2.87 74.1 25.9 273.2	High-performance liquid chromatography (HPLC)	[37]
Polystyrene (PS)	2–100	1.0	Hexaconazole Myclobutanil Triadimenol	100	- 185 0.208	Ultra-high performance liquid chromatography (UHPLC)	[45]
Polypropylene (PP)	1–10	10	Imazamox Imazapic Imazethapyr	1000	- 0.81 -	High-performance liquid chromatography (HPLC)	[49]
PE	260	1.0	Phenanthrene Tonalide Benzophenone		-	Gas chromatography-mass spectrometry (GC-MS)	[53]
PE PP Polyvinyl chloride (PVC)	<0.15	0.5	3,6-dibromocarbazole (3,6-BCZ)	500	PE: 15.3 PP: 12.3 PVC: 16.2	High-performance liquid chromatography (HPLC)	[50]
			3,6-dichlorocarbazole (3,6-CCZ)		PE: 24.8 PP: 28.5 PVC: 27.8		
			3,6-diiodo carbazole (3,6-ICZ)		PE: 118 PP: 38.2 PVC: 322		
			2,7-dibromo carbazole (2,7-BCZ)		PE: 16.6 PP: 18.3 PVC: 35.2		
			3-bromocarbazole (3-BCZ)		PE: 17.1 PP: 8.39 PVC: 17.5		
PE PS PVC PP	75–150	1.0	Fipronil	0–300	PE: 57.5 PS: 50.8 PVC: 38.3 PP: 62.7	High-performance liquid chromatography (HPLC)	[44]
PE	49–259	0.3	Trichlorobenzenes (1,2,3-TeCB, 1,3,5-TeCB, 1,2,4-TeCB) Pentachlorobenzene Hexachlorobenzene Trifluralin	100	227–333	Gas chromatography (GC)	[54]
PE PS	260 250	1.0	Atrazine/Carbendazim/DEET/Diazinon/ MCPA/Mecoprop/ Propiconazole/ Tebuconazole/ Terbutryn The mix included pharmaceutical and personal care products: Benzotriazole/Caffeine/Carbamazepine/Diclofenac/Ibuprofen/4-Nonylphenol/Tris(2-chloroisopropyl)-phosphate/Torasemide/Triclosan	5 Exceptions: phenanthrene 50 nonylphenol 30	-	Gas chromatography-mass spectrometry (GC-MS)	[55]
**Pharmaceutical and personal care products**							
Low-density polyethylene (LDPE) PS	300 250	0.4	Venzophone-3 4-methyl benzylidene camphor Ethylhexyl methoxycinnamate Octocrylene	20–200	-	High-performance liquid chromatography (HPLC)	[56]
Virgin PS Aged PS	450–1000	1.6	Oxytetracycline	20,000	Virgin PS: 1520 Aged PS: 27,500	High-performance liquid chromatography (HPLC)	[57]
Virgin PS UV-aged PS Virgin PVC UV-aged PVC	75	0.4	Ciprofloxacin	10,000	Virgin PS: 10,200 UV-aged PS: 54,800 Virgin PVC: 11,700 UV-aged PVC: 1550	Fourier-transform infrared spectroscopy (FTIR)	[58]
PE	100	2.0	Ciprofloxacin	25,000	5850	Fourier-transform infrared-attenuated total reflectance (FTIR-ATR)	[59]
PE PP PS PVC	200	-	Tylosin	5000	PE: 1670 PP: 3333 PS: 3333 PVC: 3333	Fourier-transform infrared spectroscopy (FTIR)	[60]
PE PS PVC	PE: 28–590 PS/PVC: 75	0.5	Tetracycline	5000	-	Fourier-transform infrared spectroscopy (FTIR)	[61]
Polyamide (PA) PE Polyethylene Terephthalate (PET) PS PVC PP	100–150	2.0	Sulfamethoxazole	2400	PA: 96,400 PE: 660 PET: 710 PS: 114,000 PVC: 2800 PP: 6900	High-performance liquid chromatography (HPLC)	[46]
Aged PS Aged PE	100–200	2.0	Sulfamethoxazole Sulfamethazine Cephalosporin-C	2000	Aged PS (Cephalosporin-C): 710 Aged PE (Cephalosporin-C): 720	Fourier-transform infrared spectroscopy (FTIR)	[62]
Virgin polylactic acid (PLA) Aged polylactic acid (PLA) Virgin PVC Aged PVC	PLA: 250–500 PVC: 75–150	0.4	Tetracycline	5000	PLA: 2510 Aged PLA: 5490 PVC: 960 Aged PVC:1570	Fourier-transform infrared spectroscopy (FTIR)	[63]
			Ciprofloxacin		PLA: 3190 Aged PLA: 3770 PVC: 670 Aged PVC: 850		
PE	45–48	0.2	Sulfamethoxazole Propanolol Sertraline	60	-	Ultra-high performance liquid chromatography-tandem mass spectrometry (UPLC/MS/MS)	[64]
PVC	110	0.05	17β—Estradiol 17α—Ethynylestradiol	10	-	Ultra-high performance liquid chromatography (UHPLC)	[65]
PP	450–850	2	Tonalide Musk xylene Musk ketone	5	-	High-performance liquid chromatography (HPLC)	[66]
PS	60–200	5	Triclosan	2500	-	High-performance liquid chromatography (HPLC)	[67]
PVC	Small/Large particles	0.4	Triclosan	10,000	-	Ultraviolet–visible spectrophotometry (UV/VIS)	[68]
PE PS	225 313	4	Triclosan	5800	-	Fourier-transform infrared spectroscopy (FTIR)	[69]
**Metals**							
PE	60–150	5	Copper (Cu)	500–5000	30.8	Gas chromatography (GC) inductively coupled plasma atomic emission spectrometry (ICP-AES)	[47]
PA PE PS PET PVC Poly(methyl methacrylate) (PMMA)	70 204 192 351 138 75	0.4	Cu	50–10,000	PA: 324 PE: 8.28 PS: 8.46 PET: 8.71 PVC: 6.29 PMMA: 41.0	Flame atomic absorption spectrophotometry	[70]
PE PP PMMA	290 85 6	1	Cu Lead (Pb)	20,000 100,000	PE: 2010 PP: 1570 PMMA: 4210	μ-Fourier-transform infrared spectroscopy (μ-FTIR)	[71]
PE PET PP PS PVC	<5000	0.1	Cobalt (Co)	1000	PS: 813	Fourier-transform infrared spectroscopy (FTIR)	[72]
			Zinc (Zn)		PE: 505 PVC: 634		
			Chromium (Cr)		PE: 4700 PP: 624 PS: 473 PVC: 2240		
			Cu		PE: 259 PP: 2950 PS: 358		
			Pb		PE: 2360 PET: 4930 PP: 5550 PS: 2940 PVC: 1900		
PE PP PVC PS	-	0.4	Pb	1000	13,600	Flame atomic absorption spectrophotometry (FLAAS)	[73]
Virgin HDPE Aged HPDE	-	10	Cr	5	Virgin HDPE: 0.297 Aged HPDE: 0.441	Inductively coupled plasma-mass spectrometry (ICP-MS)	[74]
			Co		Virgin HDPE: 0.018 Aged HPDE: 0.038		
			Ni		Virgin HDPE: 0.008 Aged HPDE: 0.070		
			Cu		Virgin HDPE: 0.261 Aged HPDE: -		
			Cd		Virgin HDPE: 0.0004 Aged HPDE: 0.010		
			Pb		Virgin HDPE: - Aged HPDE: 0.716		
Virgin PE PE aged on beach	4000 (Average)	10 12	Silver (Ag)	5	Virgin PE: 0.0128 Aged PE: 1.068	Collision cell–inductively coupled plasma-mass spectrometry (ICP-MS)	[75]
			Cd		Virgin PE: 0.0101 Aged PE: 0.248		
			Cr		Virgin PE: - Aged PE: 0.0933		
			Co		Virgin PE: 0.0692 Aged PE: 0.0796		
			Cu		Virgin PE: 0.100 Aged PE: -		
			Mercury (Hg)		Virgin PE: 0.170 Aged PE: 2.78		
			Ni		Virgin PE: 0.0166 Aged PE: 0.152		
High-crystallinity polyethylene (HPE) Low-crystallinity polyethylene (LPE) Chlorinated polyethylene (CPE) PVC	280	0.125–2	Cu	100–50,000	HPE: 385 LPE: 56 CPE: 3868 PVC: 431	Gas chromatography-mass spectrometry (GC-MS)	[76]
			Cd		HPE: 242 LPE: 345 CPE: 7485 PVC: 1748		
			Pb		HPE: 283 LPE: 590 CPE: 1109 PVC:2518		

## 3. Sampling and Analytical Methods

Different studies have shown several ways to perform the extraction and analysis of microplastics without a standard guideline. In order to establish procedure guidelines and reproducible analytical methods, it is extremely important for future works in microplastic exploration to provide a detailed description of the procedures, from the preparation of samples to the analysis of the results.

### 3.1. Sample Preparation

The diversity of microplastics can be seen as a challenge when it is necessary to choose the sample preparation method. Different sizes, polymer types, shapes, aging states, and possible additives in their composition are characteristics that should be considered when choosing the sampling process. The preparation of samples has been recognized as one important step to the success of the analysis required; however, it must take into account the research questions and objectives of the study [77,78].

During laboratory work, some guidelines should be followed to avoid contamination: in all experiments, the plastic material should be replaced by non-plastic, but if this is not possible, blanks are necessary for controlling whether plastic particles are created during the preparation [77].

Pre-treatment steps depend directly on the sample matrix, but it is extremely important to ensure the removal of the non-plastic materials, such as minerals and organic matter, while simultaneously extracting microplastics without any damage to the particles. For this pre-treatment step, microplastics can be separated according to their physical and chemical (digestion/extraction) characteristics [79].

Some researchers have published different methods to remove microplastics from large sediment samples, showing the potential of the Microplastic Sediment Separator (MPSS) to ensure the separation of different sizes of microplastics from sediments [80,81], with a recovery of 13–39%.

Another described approach is the use of an electrostatic separator as a complement to the MPSS, to promote a reduction of 90% in the non-polymer matrix. The release of the discharged energy by polymers is slow; on the other side, the discharge by minerals and organic particles is quite fast, and it is possible to promote this separation. The cost effectiveness of this process is one of the advantages, due to the short processing time and the fact no chemicals need to be added to promote the separation. Still, organic digestion and density separation should be performed to retain the sample fraction of microplastics in the sediments [77].

Acidic, alkaline, and enzymatic digestion are the most reported approaches to separate microplastics from real samples. Generally, the process of chemical or enzymatic digestion is widely used to destroy the organic matter in the sediments. The use of hydrogen peroxide (H_2_O_2_) has been reported as a sustainable method to extract microplastics, and it has also been recommended in an extensive guide of the European Marine Strategy Framework Directive (MFD), which also included the use of potassium hydroxide (KOH) solutions [79].

For acid digestion, the most used acids are nitric (HNO_3_) and hydrochloric (HCl) acids, with the highest capacity for degradation of 94–98% being reported for the use of nitric acid [82]. However, it is important to ensure a good choice of acids, since some reports mentioned the degradation of polymers, such as PA, with the use of strong acids [79]. For alkaline digestion, the use of sodium hydroxide (NaOH) is also a possibility, with a recovery efficiency of 90% when the solution concentration is 1 M [77].

Enzymes can also be used for the digestion of plastic samples, since they have the capacity for removing organic matter and decreasing the biological tissue [83]. In contrast to chemical digestion, in enzymatic digestion, there is no risk of degradation of the samples. However, the time necessary is a disadvantage of its application, and in large samples, it may be expensive [77]. It is important to underline the physical damages that samples can suffer during chemical digestion due to mechanical friction, or losses due to the heating of the sample [77].

Density separation is mostly used for separating microplastics from sediments after the use of chemical or enzymatic digestion. For this process, a high-concentration or saturated salt solution is added and mixed with the sample by shaking [83]. Plastics float at the surface and can be separated since they have low densities.

Several studies presented different salts according to the microplastic properties. For the removal of polymers such as PP (0.8 g/cm^3^) or PA (1.13 g/cm^3^), the use of sodium chloride (NaCl) solutions (20%) is recommended, in a temperature range of 10 to 20 °C. The use of this salt has the advantages of a low cost and low toxicity [84]. For polyvinyl chloride or polyethylene terephthalate, which have a high density, the use of sodium iodide (NaI) has been studied. However, the high cost of this product leads to an improvement in the process with a previous step with sodium chloride solutions, followed by the NaI. With this two-step process, the consumption of the NaI is reduced, making the cost of the process viable [85].

Zinc chloride (ZnCl_2_) solution has been used coupled with the process of the MPSS. This approach showed a high recovery rate of microplastics and a cheap solution. The disadvantage is the hazardous and corrosive properties of the ZnCl_2_ [84].

### 3.2. Methods for Microplastic Analysis

Visual identification, thermal degradation, and spectrometry methods have been mentioned for microplastic analysis. All the developed techniques have strong advantages, but some fragilities have also been reported.

#### 3.2.1. Visual Identification

Visual identification can be useful to characterize and quantify nano- and microplastics with an average size of 500 μm. This technique is appealing because it is simple and has a low cost; however, it is not recommended as an independent method for microplastic detection. The use of this technique may lead to an underestimated or overestimated microplastic abundance, since reports have mentioned that some microplastic particles were later identified as natural fibers and materials, misleading the determination of the precise amount of microplastics [79].

#### 3.2.2. Thermal Degradation

Pyrolysis (PYR) and thermal extraction and desorption (TED), coupled with gas chromatography (GC) and mass spectrometry (MS), are reported as destructive techniques that allow the identification of the polymer types and organic chemicals that may be associated with them [79]. The principle of these methods is based on the decomposition products of heated samples and identifies the types of additives and polymers. Thermal techniques do not change the sample, and the pre-treatment steps can be eliminated. However, some known disadvantages must be considered in the choice of the experimental method, since pyrolysis with GC-MS is limited to analyzing low quantities of samples, which makes the analysis of a large number of samples unsustainable. Further, some polymers can have similar decomposition products which may lead to a misinterpretation of the results [79].

#### 3.2.3. Spectrometry Methods

The physical characterization of microplastics, such as the number, color, and size, cannot be determined using thermal degradation techniques [86]. Spectrometry methods are also mentioned as possible methods to analyze microplastics, such as Raman spectroscopy and Fourier-transform infrared spectroscopy (FTIR). Raman spectroscopy is a non-destructive spectroscopic technique that provides specific information on high-molecular-weight polymers and leads to the identification of the microplastic particles within minutes [79,83]. The application of this technique detects particle sizes smaller than 20 μm; however, the analysis time is too long. The fluorescence of sample compounds may interfere with the polymer identification; for Raman spectroscopy, a purification step on the samples is recommended. The lack of standardization of the methods leads to different research studies with different system parameters, such as the wavelength choice of 532 nm or 785 nm [79].

FTIR is used to identify microplastics by attenuated total reflection (ATR), transmission, and reflection, leading to an exact identification of polymers based on their IR spectra. Microplastics with particle sizes larger than 300 μm can be detected, and the results can be obtained in 1 min with great precision; however, the sample has to be completely dry, and it should be isolated to avoid any interferences in the results. The fluorescence does not interfere with this technique, marking the difference between this technique and Raman spectroscopy. FTIR can provide information about the abundance and structure of the particles and can also detect information regarding the weathering of the samples. This information permits an estimation of the degree of degradation or even biodegradation based on the intensity of oxidation. Dark and opaque plastic particles can be difficult to detect, and when they are present, they can affect the analysis. The time is also a disadvantage of this process since the detection is carried out with one particle at a time, resulting in a significant amount of laboratory processing time being consumed [86].

Different sizes, colors, and shapes are characteristics of microplastics, which can affect the analyses of the samples. It is important to evaluate and adjust the procedure to the type of samples. Standardization of the methods should be the next step to make the exploration of the invasion of microplastics in the environment easier, such as its consequences and risks too.

## 4. Toxicity of Microplastics

The ingestion, accumulation, and toxicity of microplastics in different organisms have been reported and represent a current public health concern since toxicity has not yet been fully investigated. Human exposure to microplastics can occur through ingestion, inhalation, and dermal contact due to their presence in food, water, air, and consumer products [29]. Microplastics’ presence in food samples has been reported, mainly in seafood species, such as fish, shrimp, and bivalves, but also in other foods such as honey, beer, and table salt [87]. Recently, the presence of microplastics was demonstrated in the human placenta [88], blood [89], and lung tissue [90].

The effects of these particles in the organism system depend on exposure and vulnerability, and they are seen as potentially harmful. The impact on organisms is still under exploration; however, it is known that they may lead to oxidative stress, cytotoxicity, and translocation to other tissues. The persistence of microplastics in organisms can lead to chronic inflammation, which can increase the risk of cancer and may also be related to the increase in immune or neurodegenerative diseases and metabolic disturbances [10,22].

In addition to the negative impact of the dispersion of microplastics in the environment, there are several reports of hazardous ecotoxicological effects on soil organisms, such as plants and small soil invertebrates. Significant changes in biomass, tissue elemental composition, root traits, and root symbioses have been reported in the presence of different microplastics [91]. Studies have proved that microplastics can decrease the gut microbial community, reproduction, and avoidance behaviors of springtails, and promote disrupted energy metabolism, slow down the locomotor behavior, and decrease the body length of nematodes [92,93]. The ingestion of these particles can inhibit the food intake and excretion of snails and affect oxidative stress [34]. Microplastics combined with other pollutants, such as PAHs, PCBs, or metals, can promote harmful consequences for the organisms and should be a focus of research to understand the different potential combinations that may be faced currently and to define goals to minimize the impact of these emerging pollutants.

## 5. Global Microplastic Distribution: A Case Study in Europe

The global production of plastics has achieved record numbers; between the 1950s and 2015, the global production of plastics, including primary and secondary sources, achieved a total of 6.3 billion tons, of which only 9% has been recycled, 12% incinerated, and 79% stored in landfills [94]. According to the statistics about the production of and pollution promoted by plastics, it is estimated that in 2050, there will be more plastic than fish in the sea (by mass) [95,96]. However, large plastics are not the only issue that the ocean and terrestrial ecosystems are facing. There are some predictions of an estimated release from 0.8 to 2.5 million tons per year of primary microplastics ending in the oceans. At the moment, it is predicted that Asia, North America, and Europe will make major contributions to the microplastics released into the environment [19].

Despite the limited published information, in general, two main origins of microplastics are presented: land-based and ocean-based with an estimated 75–90% and 10–25% of the plastic debris in the marine environment, respectively. From the land-based contribution, it is possible to enumerate different types of sources, such as construction sites and building maintenance, municipal landfills, municipal wastewaters, application of treated sludge in agriculture, and other sources that can contribute to the incorrect disposal of plastics. However, tires and road wear particles and the synthetic fibers from laundering are considered the main sources of the land-based contribution. Tourist activities, commercial fishing, and shipping were reported by Cole et al. [3] as some of the biggest sources of microplastics. Aquaculture facilities, offshore oil or gas platforms, and cargo lost from merchant ships have also been mentioned as contributing to the increase in the presence of microplastics [3,16].

Ajuth, N. et al. (2020) [97] focused on the distribution of works about microplastics in the different continents, showing that Europe presented the largest number of studies (38%), followed by Asia with 36%, North America with 12%, and South America with 7%.

In the European context, different studies about microplastics in several countries (Spain, France, Portugal, Italy, etc.) have been reported [98].

In Spain, Filgueiras et al. [99] confirmed the widespread distribution of microplastics in the coastal sediments along with the Spanish Mediterranean coast, from Algeciras to Barcelona. This study presented the number of microplastics (MP) per kilogram of sediment dry weight (kg^−1^ d.w.) in different areas. Málaga presented 280.3 ± 164.9 MP kg^−1^ d.w. and Palma de Mallorca 45.9 ± 23.9 MP kg^−1^ d.w. For Barcelona and Denia, an abundance of 148 MP kg^−1^ d.w. and 156 MP kg^−1^ d.w., respectively, was reported [98]. Bayo et al. [100] reported a study focused on the removal of microplastics in an urban wastewater treatment plant, situated in Cartagena. The removal efficiency of the microplastics was 90.3%, decreasing from 3.20 ± 0.67 MP L^−1^ to 0.31 ± 0.06 MP L^−1^.

In France, the abundance of microplastics was studied in 40 samples from the Golf Lion. In this study, it was reported that 90% of these stations contained an average of 0.116 particles of MP per m^2^ [101]. Schmidt et al. [102] collected 43 microplastic samples in the northwestern Mediterranean Sea and showed the presence of microplastics at a concentration ranging from 0.06 to 1 MP per m^2^.

In Italy, Baini et al. [103] determined the microplastic concentration along the Tuscany coastal waters. The results showed an average concentration of 0.26 items of MP/m^3^ in the water column samples. In the surface samples, concentrations ranging from 41 to 69,161 items/km^2^ of floating microplastics were found.

Portugal is a coastal country with 1230 km of Atlantic coast and a high probability of being affected by the impact of marine litter. Prata et al. [104] reported that the wastewater treatment plants (WWTPs) in Portugal treated 550 million m^3^ of wastewater, resulting in 580 million m^3^ of treated effluent in 2010, which means that 4.1 trillion microplastics per year are released into the oceans [104]. WWTPs also produce sludge as a by-product of the treatments, and some reports proved that the main part of microplastics is retained in the sludge. Prata et al. [104] reported that, in 2013, Portugal used 105,400 tons of sludge in agriculture, of a total of 338,800 tons, and this practice led to the spread of microplastics in the environment. Studies reported that the highest microplastic concentration in Portugal is located in the center beaches, in the range between 590 and 2126 items per m^2^, followed by Lisbon and the North Region [16,104,105].

## 6. Removal Strategies

### 6.1. Existing Treatments

There are no legal limits for microplastics in the environment, and the experimental monitoring limitations are a problem. Researchers have found some difficulties in the experimental methods since there are no standardized methods to separate and analyze microplastics. In parallel, the sampling in wastewater treatment systems has some challenges that must be taken into consideration, such as the variability of flow rates during the day and the variation in pollutant concentrations [106].

Regardless of these difficulties, it is important to understand how wastewater treatments can help to decrease the presence of microplastics, because of the small size of the particles that may pass throw the different treatment steps and consequently be discharged into rivers [6,107]. Iyare et al. [108] reported several studies about the impact of WWTPs on the concentration of microplastics in the treated wastewater, considering the sampling method, identification, and concentration of microplastics in the influent and effluent of WWTPs in different locations. With the information collected from 21 studies, it was possible to conclude that with a preliminary/primary treatment, an average removal of 72% of microplastics was achieved, while with secondary and tertiary treatments, the removal stayed at an average of 88% to 94%. The activated sludge process is also responsible for the removal of 7–20% of microplastics [109]. Thus, besides studying the sedimentation of microplastics, the shapes and sizes of the particles must be considered important factors for their removal. Despite these good removal efficiencies, it is important to underline that there is a significant variation in the concentration of microplastics in the influent and effluent, which can be related to the variation in the sampling, isolation, and detection methods. Even though WWTPs are not prepared for removing microplastics, good removal efficiencies of around 98–99% have been reported; nevertheless, they may still be a considerable source of microplastics in the effluents discharged [106,110,111].

### 6.2. Advanced Treatments

Since microplastics are present in treated wastewaters and drinking waters [19,104], several studies have reported the importance of understanding the actual removal efficiencies of microplastics in conventional treatments and how to enhance their removal. Although actual water and wastewater treatments are not projected to remove microplastics, they present good removal efficiencies. Nevertheless, they should be complemented by advanced treatments to enhance microplastic removal. Researchers have been developing and testing different approaches that show possible routes that can be taken in the future. However, it is important to mention that most of the experiments were performed on a laboratory scale and still need to be tested on industrial scales.

WWTPs have been projected to remove contaminants from wastewater, and even though microplastics do not make up part of the list, they have started to be seen as an important focus for removal. Regardless of their efficient removal, WWTPs continue to be a source of microplastics in the aquatic and terrestrial environment. Several tertiary treatments have been tested successfully, although with variable efficiency: rapid sand filtration (55.6–95.0%), micro-screen filtration with disc filters (40.0–98.5%), dissolved air flotation (95.0%), and membrane bioreactors (33.3–99.9%) [112]. Complementing these treatments with new advanced treatments will be the way forward to enhance the removal of microplastics. Recently, different treatment approaches have been reported, namely, electrodeposition, electrocoagulation, and dynamic membranes [113].

The combination of biological wastewater treatment with microfiltration or ultrafiltration in a membrane bioreactor [112] presents a higher maximum efficiency (99.9%) than the conventional primary (95.0%) and tertiary treatments (98.5%), which makes it one of the most efficient technologies that should be explored [113]. Recent studies reported a removal percentage of 100% of commercial PS beads with a magnetic polyoxometalate-supported ionic liquid phase and total removal of PE, PET, and PA microplastics using magnetic carbon nanotubes [114,115].

Electrocoagulation is another advanced treatment that combines the benefits of coagulation, flotation, and electrochemistry. This method promotes the removal of particles, such as microplastics, by destabilizing the repulsive forces that keep the particles suspended in water. Then, the forces are neutralized, and the suspended particles start to aggregate, favoring their removal from the wastewater [116]. The reported advantages are the effective cost, compatibility with different particles, and sludge production minimization [117]. Perren et al. [118] demonstrated the effective removal, of around 90%, of microbeads from the water, with pH values ranging from 3 to 10, suggesting that this can be an effective method to study different microplastics.

The sol–gel method is a new treatment method that has been used to remove polymers by induced agglomeration of polymers in wastewater, forming big particle agglomerates. This is an economical process that presents good chemical stability, since the use of synthetic amorphous silica (SAS) is very common and successful as a catalyst, carrier, and adsorbent [119].

This process has been tested under alkaline and acidic catalytic conditions, and according to Zhang and Chen [117], the sol–gel method can promote the flocculation of microplastics. With the formation of big microplastic flocculates, the process of the isolation and separation of plastic particles from the water is achieved. The different types and sizes of particles, the concentration of trace contaminants, and external factors, such as pH, temperature, or pressure, are independent of the agglomeration process, being an advantage of the use of this method [117].

Recently, a new approach that involves using coated Fe nanoparticles to magnetize plastics, allowing magnetic extraction and the isolation of microplastics, was explored [120,121]. The hydrophobic surface of microplastics allows their magnetization via binding nanoparticles [121]. Shi et al. [121] reported that this approach was effectively applied to remove microplastics in environmental water bodies including river water, domestic sewage, and natural seawater, with a removal rate higher than 80%. Additionally, nanoencapsulation has been showing promising results in this area, but it needs to be further explored [122].

Dynamic membrane technology is a possible removal technique applied to the removal of microplastics. This method consists of a layer of particles deposited via permeation and pulled onto a conventional membrane, such that the deposited particles act as a secondary membrane to minimize fouling of the primary membrane to lower transmembrane pressures. It is like a cake layer on a supporting membrane. One of its major advantages is the non-existence of chemicals or the generation of by-products. This can be a widely applied method as it can be used to support membranes with a large pore mesh or other low-cost porous materials [123].

## 7. Conclusions

Microplastics are an emerging pollutant with standard analytical procedures still under development, which limits their accurate monitoring in the environmental compartments. The absence of standardization of the sampling and analytical methods is generating confusion about the real state of this contaminant.

Moreover, according to the reported studies, they interact and present the ability to sorb other contaminants, such as pesticides, pharmaceuticals, and metals. Studies have demonstrated the interactions between microplastics/contaminants and reinforced the idea of microplastics as a vector for the transport of contaminants into different ecosystems. Even though it was possible to confirm the important role that microplastics play, future works must proceed since there is an infinity of combinations and potential spreads into the environment.

The implementation of legislation limiting the concentration of microplastics would be a path to ensure that they start to be seen as a pollutant, with there also being a pressure to ensure that new, precise, and accurate methods are developed for the sampling and analysis of microplastics.

While standardization is not available, which can lead to the misidentification of microplastics, in further works, detailing all the steps (sampling, treatment, solution preparations, equipment required) should be a priority to allow future developments and improvement of the procedures and reach a more precise evaluation.

Several treatment processes have been exposed, such as sol–gel treatment, electrocoagulation, magnetic separation, and dynamic membrane technology; however, most of them have only been tested on a laboratory scale with a few microplastics. New treatment alternatives must continue to be explored and tested in real samples, where a mixture of microplastics and different contaminants is present. It is also important to approach both lab tests and the industrial scale to ensure the viability of strategies to be implemented in future wastewater treatments.

## Data Availability

Not applicable.
